# The therapeutic HIV Env C5/gp41 vaccine candidate Vacc-C5 induces specific T cell regulation in a phase I/II clinical study

**DOI:** 10.1186/s12879-017-2316-x

**Published:** 2017-03-24

**Authors:** Kristin Brekke, Maja Sommerfelt, Mats Ökvist, Anne Margarita Dyrhol-Riise, Dag Kvale

**Affiliations:** 1 Department of Infectious Diseases, Oslo University Hospital, Oslo, Norway; 2 Bionor Pharma, Oslo, Norway; 3 University of Oslo, Oslo, Norway; 4 K.G. Jebsen Inflammation Research Center, University of Oslo, Oslo, Norway

**Keywords:** HIV, Therapeutic vaccine, Immune regulation, T cell, Antibodies, Immune activation

## Abstract

**Background:**

Levels of non-neutralising antibodies (AB) to the C5 domain of HIV Env gp120 are inversely related to progression of HIV infection. In this phase I/II clinical study we investigated safety of Vacc-C5, a peptide-based therapeutic vaccine candidate corresponding to C5/gp41^732–744^ as well as the effects on pre-existing AB levels to C5/gp41^732–744^, immune activation and T cell responses including exploratory assessments of Vacc-C5-induced T cell regulation. Our hypothesis was that exposure of the C5 peptide motif may have detrimental effects due to several of its HLA-like features and that enhancement of non-neutralising anti-C5 AB by vaccination could reduce C5 exposure and thereby chronic immune activation.

**Methods:**

Thirty-six HIV patients on effective antiretroviral therapy were randomised to one of three dose levels of Vacc-C5 administered intramuscularly with Alhydrogel or intradermally with GM-CSF as adjuvant through initial immunisation and two booster periods over 26 weeks. Vacc-C5-specific AB were measured by ELISA and T cell responses by both IFN-γ ELISPOT and proliferative assays analysed by flow cytometry. Immune regulation was assessed by functional blockade of the two inhibitory cytokines IL-10 and TGF-β in parallel cultures. Non-parametric statistical tests were applied.

**Results:**

Vacc-C5 was found safe and well tolerated in all patients. Only marginal changes in humoral and cellular responses were induced, without any effect on immune activation. Overall, anti-Vacc-C5 AB levels seemed to decrease compared to pre-existing levels. Whereas Vacc-C5-specific CD8^+^ T cell proliferative responses increased after the first booster period (*p* = 0.020; CD4^+^, *p* = 0.057), they were reduced after the second. In contrast, Vacc-C5-induced T cell regulation increased after completed vaccination (*p* ≤ 0.027) and was lower at baseline in the few AB responders identified (*p* = 0.027).

**Conclusions:**

The therapeutic HIV vaccine candidate Vacc-C5 safely induced only marginal immune responses, whereas Vacc-C5-induced T cell regulation markedly increased. Our data support further attention on immune regulation during therapeutic HIV vaccination studies.

**Trial registration:**

NCT01627678.

**Electronic supplementary material:**

The online version of this article (doi:10.1186/s12879-017-2316-x) contains supplementary material, which is available to authorized users.

## Background

More than 30 years after its discovery, Human Immunodeficiency Virus type 1 (HIV) remains a global challenge with more than 36 million people infected worldwide [1]. Despite the achievements of effective antiretroviral therapy (ART), patients with access to ART control viremia but are not cured. Chronic immune activation is reduced, but persists [2] and is associated with non-AIDS morbidity and mortality [3, 4], an increasing concern in the long term care of HIV patients. Search for alternative treatment strategies are therefore warranted.

Vacc-C5 is a peptide-based therapeutic vaccine candidate that aims to induce non-neutralising antibodies (AB) to the 5th constant domain (C5) of the HIV-1 envelope glycoprotein gp120 in conjunction with part of the transmembrane glycoprotein of gp41. The presence of AB towards the C5 region has previously been shown to correlate with a slower disease progression [5–7]. Analysis of serum from treatment naïve patients has demonstrated higher levels of non-neutralising anti-Vacc-C5 AB levels in HIV natural viral suppressors with moderate viral loads (<10,000 copies/ml) than in patients with higher viral loads (>10,000 copies/ml). Furthermore, longitudinal analysis of HIV patients has revealed an inverse correlation between anti-Vacc-C5 AB levels and disease progression [8]. The C5 domain has sequence similarity to peptide binding sites on HLA molecules [9] and can bind both defined class II and class I restricted peptides, bindings that are blocked with anti-C5 monoclonal AB [10]. It has therefore been proposed that exposed C5 could amplify chronic immune activation, either directly by cross-signalling via HLA-like motifs or by presentation of peptides that facilitate alloactivation-like responses [11]. Our hypothesis was that any detrimental effects of C5 explain the inverse relationship between anti-C5 AB levels and clinical progression, and that enhancement of non-neutralising anti-C5 AB by vaccination with Vacc-C5 could reduce exposure of C5.

Against this background, a phase I/II trial was conducted with the primary objective to evaluate safety of Vacc-C5 at three dose levels administered either intradermally or intramuscularly with two different adjuvants while on effective ART. Local low dose granulocyte-macrophage colony-stimulating factor (GM-CSF) facilitates dermal dendritic cell maturation and migration to the lymph nodes for antigen presentation [12, 13] and has been used in former studies with intradermal administration of the peptide-based HIV Gag p24 vaccine Vacc-4× [14, 15] and with peptide-based cancer vaccines [16]. Alhydrogel, the only adjuvant approved and marketed for worldwide use (e.g. pneumococcal vaccine Prevnar and HPV vaccine Gardasil), was applied in the intramuscularly administered vaccine.

The secondary objectives were to explore humoral responses to Vacc-C5, HIV-associated immune activation and T cell immunogenicity. T cell responses to HIV antigens, such as C5, may be strongly regulated in chronic HIV infection [17–19], and immune regulatory mechanisms to HIV vaccine antigens may therefore be important to consider before and during therapeutic HIV vaccination [20, 21]. We have previously assessed an exploratory parameter for vaccine antigen-induced T cell regulation, based on simultaneous blockade of the two potent, soluble cytokines downregulating HIV-specific T cell proliferation, a key feature of effector T cells [22, 23]. Our approach does not intend to differentiate the mechanistic pathways of immune regulation, but rather measure the net impact on proliferative capacity of effector T cells to simplify implementation in a clinical trial setting.

This parameter of immune regulation was addressed also in the present study, and whereas only marginal cellular and humoral immune responses were induced, vaccine-specific regulation markedly increased after completed vaccination. These results may illustrate the complexity of therapeutic HIV vaccination in general and may also hopefully encourage further attention on assessments of immune regulation.

## Methods

### Study design and immunisation schedule

**Fig. 1:**
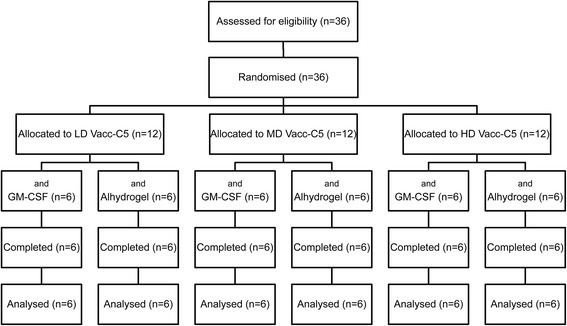
The CONSORT diagram. Patients were randomised to low (LD, 100 μg), medium (MD, 300 μg) or high dose (HD, 900 μg) with GM-CSF or Alhydrogel as adjuvant

Thirty-six HIV-infected patients (male *n* = 35; female *n* = 1) from the Outpatient Clinic, Department of Infectious Diseases, Oslo University Hospital were enrolled in this open, dose escalating phase I/II trial (Fig. 1). Patients between 18 and 55 years of age, infected for at least one year, on effective ART for the last six months with viral load <50 copies/ml, CD4^+^ T cell count >400/μl and nadir CD4^+^ count >200/μl were included. Exclusion criteria were AIDS-defining illnesses, malignancies, chronic active infections, immunosuppressive therapy or unacceptable general biochemical and hematological parameters. Pregnant or breastfeeding women were excluded.

The participants were openly and sequentially randomised to three dose groups with 12 patients in each group, and included to the allocated dose within three months after screening.

Within each dose group, patients were randomised to either intradermal injections (*n* = 6) of Vacc-C5 given 10 min after administration of the local adjuvant GM-CSF, or intramuscular injections (*n* = 6) of Vacc-C5 with Alhydrogel as adjuvant.

During the study period of 26 weeks, the participants received a total of seven doses of Vacc-C5 at either low (LD, 100 μg), medium (MD, 300 μg) or high dose (HD, 900 μg) administered at week 1, 2, 4 (primary immunisation), 12, 13 (first boost), 21 and 22 (second boost). Dose Review Group Decision meetings were regularly held to evaluate safety before the higher dose level was administered.

The study was approved by the Norwegian Regional Ethics Committee South-East and the Norwegian Medicines Agency (EudraCT 2012–000710-11) and registered at www.clinicaltrials.gov (NCT01627678). Written informed consent was obtained from all participants. The trial was carried out in accordance with the International Conference on Harmonisation Guideline for Good Clinical Practice.

### Peptide design and adjuvants

Vacc-C5 (Bionor Pharma, Oslo, Norway), a single heterodimeric peptide corresponding to the C5 domain on gp120^501–512^ and the external domain of gp41^732–744^, was supplied as sterile vials of freeze-dried powder and administered at doses of 100 μg, 300 μg or 900 μg reconstituted in water.

In the intradermal group, 0.1 ml of GM-CSF (Leukine™) (Bayer HealthCare pharmaceuticals LLC, Seattle, WA) at 0.60 mg/ml was given intradermally superficial to the left deltoid muscle 10 min before 0.1 ml of Vacc-C5 at the same site. In the intramuscular group, Alhydrogel (Apotek production & Laboratorier AB, Umeå, Sweden) was added to Vacc-C5 at a ratio of 1:1.5 in a total volume of 0.25 ml and administered in the left triceps muscle.

The vaccine was prepared by the Oslo University Hospital pharmacy on the day of administration.

### Monitoring and blood sampling

Adverse events were monitored, and clinical examination performed at each visit. Blood samples for safety (general biochemistry and hematology), CD4^+^ and CD8^+^ T cell counts and HIV RNA were analysed by approved methods. Peripheral blood mononuclear cells (PBMC) were obtained from CPT tubes (Becton Dickinson Biosciences, San Jose, CA) (BD), processed and stored at −150 °C until further analysis. Serum was processed and stored at −70 °C on site before being sent to Huntingdon Life Sciences (Huntingdon, Cambridgeshire, England) to assess anti-Vacc-C5 AB levels. Blood samples drawn at a day of vaccination were collected prior to immunisations.

### Vacc-C5-specific antibodies in serum

ELISA plates (Fisher Scientific, UK) coated with Vacc-C5 (i.e. the C5 and gp41 regions combined) were added 200 μl blocking buffer (BSA in PBS) and incubated for 2 h. 100 μl standards (Aalto Bio Reagents Sheep anti-HIV-1 gp120 AB, concentrations 0–5000 ng/mL), controls and samples diluted 1:5 were added and plates incubated for 2 h at 37 °C.

100 μl Protein G-alkaline Phosphatase Conjugate (3 μg/ml) (Calbiochem, UK) were added and plates incubated for 1 h before 100 μl Alkaline Phosphatase Yellow Substrate (Sigma-Aldrich, UK) (S-A) were added and plates incubated for 15 min. A stop solution containing Trizma Base (S-A), EDTA, NaOH and NaN_3_ was used.

Raw data were processed in Watson LIMS, using a 5PL (auto-estimate) algorithm with a weighting factor of 1/Y. Limit of detection was 390.7 ng/ml, and levels below were set to 390 ng/ml. A responder was defined as a patient with an increased AB level from baseline to end of study.

### Vacc-C5-specific T cell responses by ELISPOT

The IFN-γ enzyme-linked immunosorbent spot (ELISPOT) assay was performed on PBMC, collected at week 1 (baseline), 6, 15 and 26 (end of study), as instructed by the manufacturer (Mabtech, Nacka Strand, Sweden). The plates were washed with sterile PBS, conditioned with albumin-containing PBS and incubated at room temperature for 30 min. Cryopreserved PBMC were thawed and rested overnight before stimulated in triplicate wells with pooled Vacc-C5 15-mer overlapping peptides offset by 1 amino acid at 2 μg/ml/peptide; 200,000 PBMC/well in 200 μl medium (Gibco AIM V, Invitrogen/Life technologies, San Diego, CA). Staphylococcal enterotoxin B (SEB)-stimulated (S-A) and unstimulated cells were positive and negative controls, respectively. The plates were incubated overnight at 37 °C and 5% CO_2_ before they were washed and 7-B6–1-ALP conjugate added. After 2 h of incubation at room temperature the plates were washed and the substrate solution added. Spots developed for 12 min before the plates were washed extensively in tap water to stop the process.

IFN-γ spot forming units (SFU) were counted by an AID ELISPOT reader with AID ELISPOT v5 Scanner software (AID GmbH, Strassberg, Germany). Net SFU were determined by subtracting corresponding negative controls for each sample. No cut-off for positive response was defined. Median values of triplicates were used for analysis, and data given as SFU per 10^6^ input cells. All four time-points for a given patient were run on the same day to avoid inter-assay variation.

### T cell proliferation and regulation

Fifteen patients were selected for T cell proliferation and regulation assays at week 1, 6, 15 and 26. These included the six patients with a specific T cell ELISPOT responder profile at some time-point, defined by at least twice the negative control, regardless of their AB responses. Additionally, nine patients with varying AB responses were selected. They had either an increase in Vacc-C5 AB from baseline to end of study (*n* = 4), an increase in AB at some time-point (*n* = 3) or no AB response at all (*n* = 2). Every dose group (*n* = 6: 3: 6, LD: MD: HD, respectively) and both adjuvants (GM-CSF *n* = 6; Alhydrogel *n* = 9) were represented.

Cryopreserved PBMC were thawed and rested overnight before they were labelled with carboxyfluorescein succinimidyl ester (CFSE) (Invitrogen Molecular Probes, Eugene, OR) at 2 μM for 5 min and subsequently stimulated with pooled overlapping Vacc-C5 15-mer peptide panels at 2 μg/ml/peptide. SEB (S-A) at 0.5 μg/ml was used as positive control. 250,000 PBMC were cultured in 200 μl serum-free culture medium (Gibco AIM V, Invitrogen/Life technologies, San Diego, CA) at 37 °C and 5% CO_2_ in 96-well tissue-culture plates (Nunc, Roskilde, Denmark). At day 6, cells were harvested and stained with CD3 V450, CD8 APC-H7 and to exclude nonviable cells, 7-aminoactinomycin (7AAD) (BD). Vacc-C5-specific proliferative T cell responses were defined as percentages of live (7AAD^−^) CFSE^dim^ CD8^+^ or CD4^+^ (i.e. CD8^−^) CD3^+^ T cells. The cut-off for the proliferated CFSE^dim^ T cell subset was set by median fluorescence intensities equal to or below the second proliferated generation. Net responses were found by subtracting background proliferation in the corresponding unstimulated control cultures. All four time-points for a given patient were run on the same day.

Inhibitory monoclonal antibodies (mAbs) to soluble interleukin-10 (IL-10) and transforming growth factor-β (TGF-β) were added to parallel antigen and control cultures at 10 μg/ml final concentration according to the manufacturer’s instructions (anti-IL-10 clone 23,738, anti-TGF-β clone 1D11; R&D Systems Europe, Abingdon, UK). Antigen-induced cytokine-mediated net regulation was defined as the difference in proliferative responses between antigen cultures with and without mAbs to IL-10 and TGF-β. Fold increase was the ratio between Vacc-C5 proliferative responses with and without blocking mABs.

### T cell activation markers

Cryopreserved PBMC from all 36 patients collected at week 1, 6, 15 and 26 were thawed and rested overnight before they were stained with CD3 V450, CD8 APC-H7, HLA-DR APC and CD38 PE (BD). Activation was given as percentages of HLA-DR^+^CD38^+^ CD8^+^ or CD4^+^ (i.e. CD8^−^) CD3^+^ T cells.

For both T cell proliferation and activation markers flow cytometry data were obtained with BD FACS Canto II™ with BD Diva software v6.1.

### Analyses of soluble markers

The soluble pro-inflammatory chemokine interferon-gamma inducible protein 10 (IP-10) and cytokine IL-6 were analysed in snap frozen serum from all 36 patients at week 1, 6, 15 and 26 by Quantikine ELISA kits (R&D Systems, Abingdon, England) according to the instructions by the manufacturer. IP-10 samples were diluted 1:5. All samples for each individual were run in duplicates on the same plate.

### Statistical methods

Statistical analyses were performed by Statistica v7 (StatSoft, Tulsa, OK) using non-parametrical statistics. Mann-Whitney U test or Kruskal-Wallis was applied to compare two or more groups. Wilcoxon matched pairs test was used for dependent variables, Spearman rank for correlations and Fisher exact test to test proportional differences. All data are presented as medians (interquartile ranges, IQR). *p*-values <0.05 were considered significant. Graphics were done by GraphPad Prism v6 (GraphPad Software, La Jolla, CA).

## Results

### Safety and tolerability

All 36 patients completed the study. Their baseline characteristics are presented in Table 1. For more details, see Table 2. Vacc-C5 was safe and well tolerated, although many patients in the intradermal vaccination group experienced transient (seconds) pain/discomfort at the injection site during administration. No serious adverse event related to study drug was reported, and no significant changes in clinical status, vital signs, laboratory parameters, HIV RNA, CD4^+^ or CD8^+^ T cell counts were observed.

**Table 1:** Cohort characteristics

	All (*n* = 36)	Low dose (*n* = 12)	Medium dose (*n* = 12)	High dose (*n* = 12)
Age (years)	44.0 (38.5–49.7)^a^	45.2 (37.7–52.1)	46.3 (37.2–49.7)	42.7 (40.0–45.7)
Time since diagnosis (years)	5.2 (3.1–9.8)	4.9 (2.7–7.1)	5.0 (3.1–10.1)	8.7 (4.1–15.8)
Time on ART (years)	2.3 (1.4–3.9)	2.3 (1.6–2.7)	2.6 (1.7–4.1)	1.9 (1.3–6.6)
Nadir CD4 (cells/μl)	290 (234–360)	341 (265–396)	283 (234–343)	283 (227–358)
CD4 (cells/μl)	626 (529–758)	656 (572–756)	560 (526–679)	654 (557–831)
CD8 (cells/μl)	1051 (794–1250)	1109 (795–1223)	1032 (668–1282)	986 (805–1234)
HIV RNA (copies/ml)	0^b^(0 - <20)	0 (0 - <20)	0 (0 - <20)	0 (0 - <20)

No differences between the groups

^a^Median (interquartile range)

^b^0 implies undetectable PCR product

**Table 2:** Detailed cohort characteristics

	All (*n* = 36)	LD GM-CSF (*n* = 6)	LD Alhydrogel (*n* = 6)	MD GM-CSF (*n* = 6)	MD Alhydrogel (*n* = 6)	HD GM-CSF (*n* = 6)	HD Alhydrogel (*n* = 6)
Age (years)	44.0 (38.5–49.7)	45.2 (39.7–50.5)	44.4 (35.8–54.5)	37.2 (27.8–46.9)	49.7 (45.7–50.8)	44.0 (41.5–46.0)	40.1 (38.0–45.3)
Time since diagnosis (years)	5.2 (3.1–9.8)	3.9 (2.2–6.3)	5.9 (3.0–7.29	4.1 (2.8–11.0)	6.4 (3.4–9.1)	5.9 (3.2–15.8)	9.8 (4.6–15.9)
Time on ART (years)	2.3 (1.4–3.9)	2.0 (1.4–2.8)	2.5 (1.7–2.6)	2.3 (1.3–2.7)	3.3 (1.9–4.8)	1.3 (1.1–1.5)	4.8 (2.0–7.4)
Nadir CD4 (cells/μl)	290 (234–360) (*n* = 35)	318 (230–475)	357 (268–366)	283 (269–295) (*n* = 5)	275 (234–343)	258 (228–380)	302 (219–340)
CD4 (cells/μl)	626 (529–758)	679 (580–1006)	656 (563–728)	610 (546–713)	529 (498–596)	654 (624–840)	706 (514–821)
CD8 (cells/μl)	1051 (794–1250)	1178 (798–2268)	1002 (764–1173)	921 (345–1256)	1104 (795–1308)	905 (658–1040)	1195 (932–1258)
HIV RNA (copies/ml)	0^b^ (0 - <20)	0 (0 - <20)	0 (0–0)	0 (0 - <20)	0 (0 - <20)	0 (0–0)	0 (0 - <20)
PreART CD4 (cells/μl)^a^	341 (210–845) (*n* = 34)	358 (211–845)	360 (268–840)	295 (210–398) (*n* = 5)	301 (220–480)	306 (226–454) (*n* = 5)	370 (266–840)
PreART RNA (copies/ml)^a^	7.3 × 10^4^ (1.7 10^2^–7.3 10^5^) (*n* = 35)	8.0 × 10^4^ (1.2 10^4^–1.0 10^5^)	3.3 × 10^4^ (1.7 10^2^–7.2 10^5^)	1.5 × 10^5^ (2.6 10^4^−3.4 10^5^) (*n* = 5)	1.1 × 10^5^ (7.3 10^4^–4.3 10^5^)	4.8 10^4^ (1.7 10^4^–3.0 10^5^)	3.2 × 10^4^ (2.3 10^3^–7.3 10^5^)
Anti-Vacc-C5 AB (ng/ml)	1433 (390–4678)	390 (390–1934)	1341 (390–2396)	723 (390–4894)	1394 (458–5282)	1239 (390–4401)	7309 (3280–9772)

Median (interquartile range)

^a^Median (min-max)

^b^0 implies undetectable PCR product

### Anti-Vacc-C5 antibodies

**Fig. 2:**
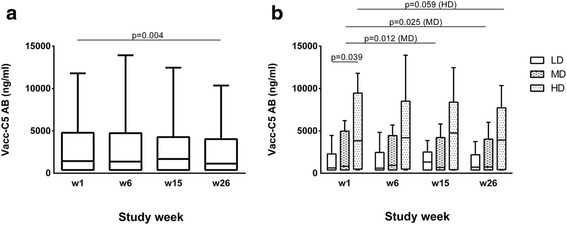
Anti-Vacc-C5-specific antibodies (AB) in serum. **a** AB levels for the total cohort at week 1 (baseline), 6 (after initial immunisation), 15 (after1^st^ boost) and 26 (after 2nd boost, end of study). Data are given as medians, interquartile and overall ranges. The change from baseline to end of study is indicated (Wilcoxon matched pairs test). **b** AB levels for the three dose groups at week 1, 6, 15 and 26. LD = low, MD = median and HD = high dose. Data are given as medians, interquartile and overall ranges. Changes within each dose group with *p*-values less than 0.10 are indicated (Wilcoxon matched pairs test)

Anti-Vacc-C5 AB were detected in 24 patients (67%) at baseline. AB levels were generally low (median 1433 ng/ml), but comparable to previous assessments (with another ELISA assay) in 102 cross-sectional sampled HIV patients on ART (median 1160 ng/ml) where 80% of the sera carried <10,000 ng/ml (data not shown). Despite randomisation, AB levels were significantly higher in the HD group compared to the LD group at baseline (Fig. 2b), but vaccine induced changes were evaluated relative to the individual’s own baseline AB level in the following.

From baseline to end of study, AB levels slightly decreased in the entire vaccinated group (*p* = 0.004) (Fig. 2a). When data for both adjuvants were combined in dose groups, AB levels were reduced in the MD (*p* = 0.025) and possibly the HD group (*p* = 0.059) whereas no changes were observed in the LD group (*p* = 0.327) (Fig. 2b).

Changes in the anti-Vacc-C5 AB levels over time did not differ between the intradermal and the intramuscular group (data not shown). However, when we divided the cohort further into groups based on both dose and administration, AB responses tended to decrease in the intradermal HD group (*p* = 0.068).

A baseline anti-Vacc-C5 AB limit of 2000 ng/ml was set according to subanalysis of a previous trial where patients with higher pre-existing levels (> 2000 ng/ml adjusted for assay differences) of anti-Vacc-C5 AB experienced improved viral outcome following Vacc-4× immunisation compared to placebo [24]. Patients with baseline anti-Vacc-C5 AB levels >2000 ng/ml (*n* = 15) showed a significant decrease in AB levels (*p* = 0.003), whereas no change was observed in patients with levels <2000 ng/ml (*n* = 13) (*p* = 0.859) (data not shown). Participants with undetectable AB levels throughout the study (*n* = 8) were excluded. Apart from the baseline AB levels, the two groups were comparable.

Even if the overall changes in anti-Vacc-C5 AB levels were small, individual differences were found. From baseline to end of study, increased AB responses were seen in six (17%) of the 36 vaccinated patients, a modest increase of 5–65% (median 26%) compared to their baseline levels. Four out of these six patients had detectable baseline levels. The six AB responders represented all three dose groups (*n* = 3: 1: 2, LD: MD: HD, respectively), and four had received intramuscular injections. Anti-Vacc-C5 AB responders (*n* = 6) and non-responders (*n* = 30) were comparable in all baseline characteristics except for the CD4^+^/CD8^+^ T cell ratio (median 0.50 [IQR 0.38–0.59] and 0.69 [0.57–0.93], respectively) (*p* = 0.046).

### IFN-γ ELISPOT responses to Vacc-C5

**Fig. 3:**
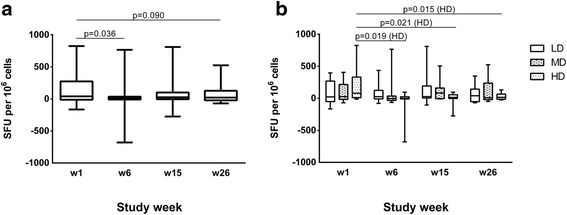
Vacc-C5-specific IFN-γ responses. **a** IFN-γ responses for the total cohort at week 1 (baseline), 6 (after initial immunisation), 15 (after1^st^ boost) and 26 (after 2nd boost, end of study). Data are given as medians, interquartile and overall ranges. Changes with *p*-values less than 0.10 are indicated (Wilcoxon matched pairs test). **b** IFN-γ responses for the three dose groups at week 1, 6, 15 and 26. LD = low, MD = median and HD = high dose. Data are given as medians, interquartile and overall ranges. Significant changes within each dose group are indicated (Wilcoxon matched pairs test)

At baseline, no significant differences in Vacc-C5 IFN-γ responses were observed between any of the three dose groups (Fig. 3b) or between the two modes of administration (data not shown).

Only small changes in IFN-γ producing cells were seen throughout the study, with a trend towards an overall decrease in SFU (*p* = 0.090) (Fig. 3a). A significant reduction was found in the HD group (*p* = 0.015) (Fig. 3b) and the HD Alhydrogel subgroup (*p* = 0.028), whereas we observed no changes within the LD or MD groups. Analysing all vaccinated, the Alhydrogel and GM-CSF groups were comparable (data not shown).

Nevertheless, 13 (36%) of the study population showed an increase in IFN-γ producing cells in response to Vacc-C5 stimulation from baseline to end of study (*n* = 5: 6: 2, LD: MD: HD, respectively). These responders were characterised by a significantly lower CD8^+^ count at baseline as compared to the non-responders (*p* = 0.005) and a tendency of higher CD4^+^/CD8^+^ T cell ratio (*p* = 0.055) (data not shown).

### Vacc-C5-specific proliferative CD8^+^ and CD4^+^ T cell responses

**Fig. 4:**
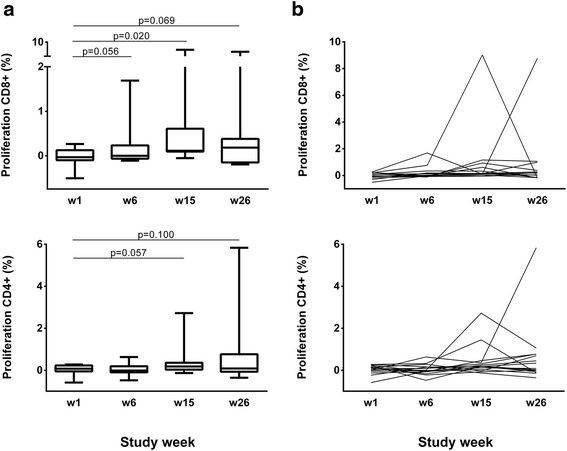
Vacc-C5-specific proliferative T cell responses. **a** Responses at week 1, 6, 15 and 26 in CD8^+^ (*upper panel*) and CD4^+^ (*lower panel*) T cell subsets. Data are given as medians, interquartile and overall ranges. Changes with *p*-values 0.10 or less are indicated. **b** Individual responses at week 1 (baseline), 6 (after initial immunisation), 15 (after1^st^ boost) and 26 (after 2nd boost, end of study) in CD8^+^ (*upper panel*) and CD4^+^ (*lower panel*) T cell subsets

From baseline to end of study, Vacc-C5-specific proliferative T cell responses tended to increase in the CD8^+^ T cell subset (*p* = 0.069). After the first booster period, CD8^+^ T cell responses increased significantly (*p* = 0.020), but were subsequently reduced after the second booster period (Fig. 4a, upper panel).

In general, small changes in proliferative T cell responses were seen, although some patients developed strong responses at different time points (Fig. 4b). At end of study, improved responses in both T cell subsets were found in nine patients (60%) including four (67%) of the six with enhanced ELISPOT responses at some time point.

As a validation of the T cell assays, we noted that the overall changes in Vacc-C5 specific responses by ELISPOT were positively correlated with the overall changes in proliferative responses from baseline to end of study (CD8^+^ *r* = 0.67, *p* = 0.006; CD4^+^ *r* = 0.51, *p* = 0.050) (data not shown).

### Vacc-C5-induced immune regulation

**Fig. 5:**
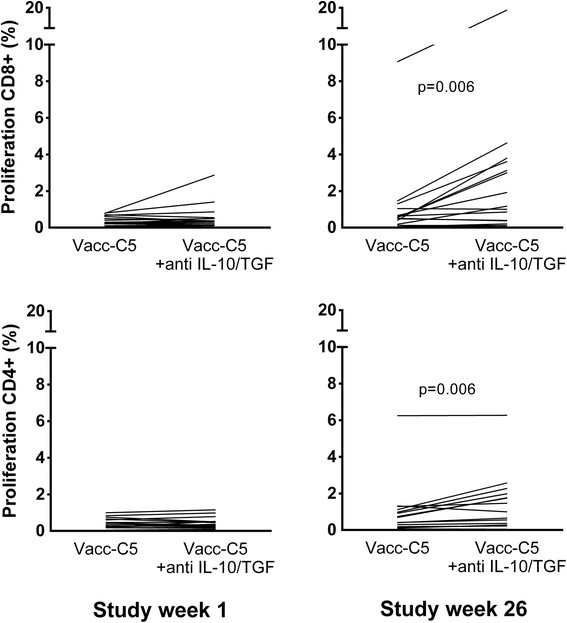
Vacc-C5-specific proliferative T cell responses without and with blockade of IL-10 and TGF-β in CD8^+^ (*upper panel*) and CD4^+^ (*lower panel*) T cell subsets at week 1 (*baseline*) (*left panel*) and week 26 (end of study) (*right panel*). Significant differences are indicated (Wilcoxon matched pairs test)

**Fig. 6:**
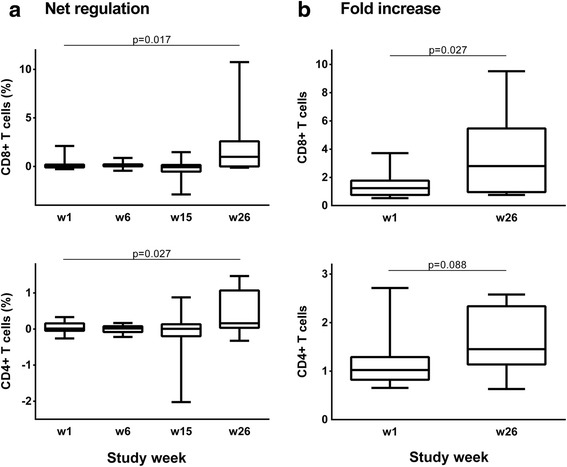
Vacc-C5-induced T cell regulation. **a** Net regulation at week 1 (baseline), 6 (after initial immunisation), 15 (after1^st^ boost) and 26 (after 2nd boost, end of study) in CD8^+^ (*upper panel*) and CD4^+^ (*lower panel*) T cell subsets. The change from baseline to end of study is indicated (Wilcoxon matched pairs test). **b** Net regulation as fold increase at week 1 and 26. The change from baseline to end of study is indicated (Wilcoxon matched pairs test)

No Vacc-C5-induced cytokine-mediated T cell regulation was detected at baseline (Fig. 5, left panel). In contrast, there was a significant difference in Vacc-C5-specific proliferative responses between cell cultures with and without blockade of IL-10 and TGF-β after vaccination (*p* = 0.006, both T cell subsets) (Fig. 5, right panel). Vacc-C5-induced cytokine-mediated net regulation increased in both the CD8^+^ (*p* = 0.017) and CD4^+^ (*p* = 0.027) T cell subsets, mainly after the last booster period (Fig. 6a). Overall, there was a three-fold increase in regulation in the CD8^+^ T cell subset (*p* = 0.027) (Fig. 6b). Notably, no correlations between proliferation and regulation were seen at end of study.

Five out of the six who increased in anti-Vacc-C5 AB were among the 15 selected for T cell proliferation and regulation assays. Interestingly, these five AB responders had a significantly lower baseline regulation in the CD8^+^ subset as compared to the ten non-responders (*p* = 0.027) (data not shown).

### T cell activation markers HLA-DR and CD38

Immune phenotyping was performed on unstimulated PBMC from all patients. Some differences between groups were found at baseline as the fraction of CD8^+^ T cells expressing HLA-DR/CD38 was higher in the Alhydrogel group (median 14.8% [IQR 11.8–18.3%]) than in the GM-CSF group (11.5% [9.4–14.2%]) (*p* = 0.037). However, from baseline to end of study, no changes in T cell activation markers expressed as HLA-DR/CD38 were observed.

### Soluble markers IP-10 and IL-6

To further investigate the possible influence of Vacc-C5 on immune activation, analyses of serum IP-10 and IL-6 were carried out, representing soluble markers that have been associated with immune activation in HIV infection. In the total cohort, the levels of the pro-inflammatory chemokine IP-10 were strongly associated with the activation marker HLA-DR/CD38 on CD8^+^ T cells at all four time points (*r* = 0.37–0.52, p = ≤ 0.025). In contrast, no correlations were found for IL-6 (data not shown). No differences between the dose groups or modes of administration were seen for IP-10 and IL-6 levels at baseline. Within the different dose and adjuvant groups there were no changes in the two parameters throughout the study period (data not shown).

## Discussion

In this phase I/II trial the therapeutic vaccine candidate Vacc-C5 was administered over six months with initial immunisation and two booster periods in the presence of ART. The primary objective was to evaluate safety of Vacc-C5 at three dose levels with two different adjuvants, while the secondary objectives were to explore humoral responses to Vacc-C5, HIV-associated immune activation and T cell immunogenicity. Immune regulation of T cell proliferative responses was an optional exploratory objective. Vacc-C5 was well tolerated, and all 36 patients completed the study. Despite rather low pre-existing anti-Vacc-C5 AB levels at baseline, only marginal changes in humoral responses to Vacc-C5 were found. Overall, both AB levels and T cell IFN-γ ELISPOT responses decreased throughout the study. The two modes of administration were comparable, with no significant differences in Vacc-C5-induced responses between them. The higher doses appeared disadvantageous as AB levels declined in the MD group, and a reduction in IFN-γ responses was seen in the HD group.

Although intra- and inter-assay variations might cause some of the minor changes in anti-Vacc-C5 AB levels, the main conclusion is that marginal changes were induced with the doses, schedule and adjuvants tested. It should be noted that Vacc-C5 has been shown to induce robust humoral responses in two classical animal models; in sheep using Alhydrogel and in rabbits using rh-GM-CSF as adjuvants (Bionor Pharma, data on file). Although not tested in HIV seronegative volunteers for regulatory and ethical reasons, Vacc-C5 was expected to induce humoral responses in humans as well. Moreover, if consistent enhancement of Vacc-C5 AB levels had been observed, additional characterisation of Vacc-C5-specific Ig isotype and subclass responses could have been assessed, although no reference to such information is available from reports on anti-C5 AB levels and slow disease progression [5–7].

Despite the minimal changes in anti-Vacc-C5 AB levels, some interesting data regarding T cell responses were observed. It should be noted that the T cell responses to Vacc-C5 were generated by pooled peptides representing the Env vaccine heterodimer, and did not differentiate responses between the two short Env regions. Overall, Vacc-C5 induced only small changes in cellular responses from baseline to end of study. After the first booster period, however, CD8^+^ T cell responses significantly increased, but were subsequently reduced after the second booster period. Strikingly, Vacc-C5-induced cytokine-mediated immune regulation was the only parameter that markedly increased during the trial. We assessed a parameter for regulation by blocking the two inhibitory cytokines IL-10 and TGF-β in Vacc-C5-stimulated cell cultures. In previous studies, this parameter has been associated with rapid disease progression [25] and inconsistent boosting effects of the HIV p24-based Vacc-4× [22]. In a recent study, where Vacc-4× was administered intranasally, immune regulation could explain most cases of T cell anergy towards HIV p24 as well as the variable final responses [23]. In the present study, increased immune regulation might partially explain the coinciding declining T cell responses after the second booster period (Figs. 4a and 6a) and possibly the lack of anti-Vacc-C5 AB responses as well. Our data find support in a recent primate prime and boost vaccination model which has shown that a SIV vaccine can induce suppressor cells and thereby dampen protective CD8^+^ T cell responses [26].

Immune regulation may be quantified in different ways. In previous papers [22, 23, 25], we have defined antigen-induced cytokine-mediated regulation as a parameter which also included control cultures with and without mAbs to IL-10 and TGF-β. Net regulation is a more simplified assessment, as reported by others [27] and illustrated in Fig. 5. Net regulation might be easier to comprehend, and is probably a more precise term to use in the discussions of this phenomenon. A variant of net regulation is fold increase, i.e. the ratio between proliferative responses in stimulated cultures with and without blockade. Here we explored immune regulation in either way with generally identical results.

Our approach is supported in part by Brockman et al. [27] who reported that blockade of the IL-10 pathway increased proliferative T cell responses to HIV antigens in viremic patients, whereas elite controllers and patients on effective ART failed to respond to the blockade. After immunisation with Vacc-C5, we found that blocking of both IL-10 and TGF-β significantly increased the proliferative capacity to Vacc-C5 in these non-viremic patients. We have previously shown that this parameter of T cell regulation is HIV antigen-specific and not an inhibitory response to T cell activation in general. In an HIV positive individual, regulation may be high to one specific HIV antigen but low or absent to other HIV antigens and non-HIV antigens. Moreover, regulation induced by different HIV antigens are not correlated in a particular sample [25, 28]. Therefore, it is likely that the regulation induced by Vacc-C5 is vaccine-specific and not a global T cell regulation induced by the vaccine.

We chose to assess regulation by blocking two inhibitory cytokines and quantify the total inhibitory effect on Vacc-C5-specific T cell proliferation. Since proliferation is a fundamental feature of effector T cells, the impact of regulation on T cell proliferative capacity may provide additional information, relevant for choosing patients expected to benefit from therapeutic vaccination.

In the era of an HIV cure, there has been a renewed interest in immunotherapy [29, 30]. Efficient HIV-specific CD8^+^ cells able to eliminate reactivated latently infected cells are essential to put HIV into remission [31], but these cells are hampered in chronic HIV infection [32, 33]. Immunotherapy may therefore be required for an effective functional HIV cure, but a possible challenge may be induction of immune regulation.

Both proliferative responses and immune regulation differed considerably from one patient to another throughout the study. Consistent with previous results [22, 23], our data highlight the importance of individualised immunotherapy. Moreover, they support the view that both proliferative responses and regulation should be monitored in therapeutic immunisation trials, because T cell proliferation and immune regulation induced by a defined HIV antigen are generally not correlated [25, 28].

This dose escalation study, with safety as primary objective, has limitations, particularly small size of each adjuvant group. Regardless, it was possible to analyse secondary endpoints in subgroups, but with the risk of type II statistical errors. Moreover, the study was planned and approved with no unimmunised or adjuvant only control groups included. Another limitation may be the selection of patients for proliferative assays. Although proliferation and regulation were not tested in all participants, these patients were comparable to the rest of the cohort regarding clinical characteristics, dose and administration as well as AB levels and IFN-γ ELISPOT responses at baseline and end of study. The regulatory assays could have been expanded to explore the individual contribution by the two inhibitory cytokines and to include other well characterised regulatory pathways by blockade of T cell receptors like CTLA-4 and PD-1. Still, all the above mentioned regulatory pathways are recently addressed by our group, and the combined blockade of IL-10 and TGF-β was found to best identify net regulation induced by Env antigens [28]. Finally, more direct methods to assess regulation might have provided even more definitive data, e.g. separation of T regulatory cells by cell sorting and add-back experiments with the sorted cells. However, these in-depth analyses would require substantial PBMC and resources unrealistic to implement in a clinical trial setting.

The development of Vacc-C5 was based on the hypothesis that the C5 region is involved in chronic immune activation and that blocking this domain with non-neutralising AB may lead to reduced immune activation and disease progression [10, 11]. However, in this study including patients on ART, increased levels of anti-Vacc-C5 AB were not induced, and the activation markers HLA-DR/CD38 and the two chosen pro-inflammatory mediators IP-10 and IL-6 remained unchanged. Since the C5 domain shows sequence similarity to HLA-DR, the HLA phenotype of study participants may potentially have influenced the results. Finally, it might be possible that Vacc-C5 administered with another adjuvant or with a carrier molecule could have generated AB with effects on HIV-associated immune activation.

## Conclusions

In this study investigating safety and immunogenicity of the therapeutic vaccine candidate Vacc-C5, only marginal immune responses were safely induced, whereas vaccine-specific regulation clearly increased after completed vaccination. It is said that we learn more from failure than success (Samuel Smiles 1812–1904, Scottish author and reformer). Despite the absence of enhanced immune responses, this study might still provide insights to improve future therapeutic vaccine designs and immunotherapy and encourage further focus on immune regulation in therapeutic vaccination.

## Additional file

Additional file 1: Supporting data. (XLSX 16 kb)

## Abbreviations

7-AAD: 7-aminoactinomycin
AB: Antibodies
ART: Antiretroviral therapy
CFSE: Carboxyfluorescein succinimidyl ester
GM-CSF: Granulocyte-macrophage colony-stimulating factor
IL: Interleukin
LD, MD, HD: Low dose, medium dose, high dose
mAb: Monoclonal antibody
PBMC: Peripheral blood mononuclear cells
SFU: Spot forming units
TGF-β: Transforming growth factor-β
